# *In-Vitro* Antibacterial Properties of Crude Aqueous and *n*-Hexane Extracts of the Husk of *Cocos nucifera*

**DOI:** 10.3390/molecules16032135

**Published:** 2011-03-03

**Authors:** Taiwo Adesola Akinyele, Omobola Oluranti Okoh, David Ayinde Akinpelu, Anthony Ifeanyi Okoh

**Affiliations:** 1 Applied and Environmental Microbiology Research Group (AEMREG), Department of Biochemistry and Microbiology, University of Fort Hare, P/Bag X1314, Alice 5700, South Africa; E-Mail: taiwoakinyele@yahoo.com; 2 Department of Chemistry, University of Fort Hare, Alice 5700, South Africa; E-Mail: ookoh@ufh.ac.za; 3 Department of Microbiology Obafemi Awolowo University, Ile Ife 220005, Nigeria; E-Mail: dakinpel@yahoo.com

**Keywords:** *C. nucifera*, *Vibrio* specie, antibacterial, *n*-hexane extract, aqueous extract

## Abstract

The increasing numbers of cases of antibiotic resistance among pathogenic bacteria such as *Vibrio* species poses a major problem to the food and aquaculture industries, as most antibiotics are no longer effective in controlling pathogenic bacteria affecting these industries. Therefore, this study was carried out to assess the antibacterial potentials of crude aqueous and *n*-hexane extracts of the husk of *Cocos nucifera* against some selected *Vibrio* species and other bacterial pathogens including those normally implicated in food and wound infections. The crude extracts were screened against forty-five strains of *Vibrio* pathogens and twenty-five other bacteria isolates made up of ten Gram positive and fifteen Gram negative bacteria. The aqueous extract was active against 17 of the tested bacterial and 37 of the *Vibrio* isolates; while the *n*-hexane extract showed antimicrobial activity against 21 of the test bacteria and 38 of the test *Vibrio* species. The minimum inhibitory concentrations (MICs) of the aqueous and *n*-hexane extracts against the susceptible bacteria ranged between 0.6–5.0 mg/mL and 0.3–5.0 mg/mL respectively, while the time kill study result for the aqueous extract ranged between 0.12 Log_10_ and 4.2 Log_10_ cfu/mL after 8 hours interaction in 1 × MIC and 2 × MIC. For the *n*-hexane extract, the log reduction ranged between 0.56 Log_10_ and 6.4 Log_10_ cfu/mL after 8 hours interaction in 1 × MIC and 2 × MIC. This study revealed the huge potential of *C. nucifera* extracts as alternative therapies against microbial infections.

## 1. Introduction

Medicinal plants contain large varieties of chemical substances with important therapeutic properties that can be utilised in the treatment of human diseases. Consequently, there is the increasingly justified assumption which claims that traditional medicine is cheaper and more effective than modern medicine. The studies of medicinal plants used as folklore remedies have therefore attracted immense attention in the scientific world in an attempt to find possible solutions to the problems of multiple resistances to the existing synthetic and conventional antimicrobials. The discovery of antibiotics had eradicated the infections that once ravaged the humankind, but their indiscriminate use has led to the development of multidrug-resistant pathogens [1].

*Vibrios* are Gram-negative, curved, rod-shaped bacteria that are natural inhabitants of the marine environment [2]. The US Centre for Disease Control and Prevention (CDC) estimates that 8,028 *Vibrio* infections and 57 deaths occur annually in the United States [3]. Transmission of *Vibrio* infections is primarily through the consumption of raw or undercooked shellfish or exposure of wounds to warm seawater [4,5]. The most common clinical presentation of *Vibrio* infection is self-limited gastroenteritis, though wound infections and primary septicemia may also occur [5]. Patients with liver disease are at particularly high risk for significant morbidity and mortality associated with these infections [6]. 

Many cases of *Vibrio* associated gastroenteritis are under-recognized due to application of inadequate diagnostic procedures [7]. The enterotoxin produced by these *Vibrio* strains causes copious, painless, watery diarrhea leading to vomiting, severe dehydration, and even death if treatment is not prompt [8]. Early detection and initiation of treatment of these infections are very important, particularly for cholera and invasive *Vibrio* infections which have high mortality potentials [9]. The CDC in 2005 estimated that the average annual incidence of all *Vibrio* infections increased by 41% between 1996 and 2005. Treatments such as antibiotic injections, aggressive wound therapy and supportive care have been adopted over the years, but persistent resistance and immunocompromising conditions recorded among patients with this infection calls for immediate attention and a need to search for more potent and new antimicrobial compounds of natural origin to combat the activities of these pathogens, which forms the basis for this research. 

*Cocos nucifera* (coconut) belongs to the family Aracaceae. The plant is mainly used as a staple food crop, and a source of wood and handicrafts, among many other uses, and is thought by many to be the world’s most useful plant and medicinal plant in tropical and subtropical countries [10]. *C. nucifera is* found throughout the tropics, where it is interwoven into the lives of the local people [10]. Esquenazi *et al*. [11] in their studies reported that in the traditional medicine in northeastern Brazil, coconut husks have been used for the treatment of diarrhea and arthritis.

Nowadays, coconut oil, obtained from the fruit of coconut palm, has been relegated mainly to non food uses in the developed countries but retains its importance for traditional uses in producing countries [10]. Coconut oil has been confirmed to possess antimicrobial, antiviral and antiprotozoal activities [12,13]. Though the antibacterial potential of the husk of *C. nucifera* has been reported before [11,14], these studies have not been elaborate enough and have covered very few bacterial strains. For example, Esquenazi *et al*. [11] used only one bacterial species (*Staphylococcus aureus*), which though pathogenic is not a known diarrheic pathogen. Also, Srinivas *et al*. [14] relied only on five bacterial species. There is need for a more detailed assessment of the antibacterial potential of the husk of *C. nucifera* against a wide panel of bacterial species including referenced, environmental and clinical strains which is the focus of this current report. In this paper, we report on the antibacterial properties of the aqueous and n-Hexane extracts of the husk of *C. nucifera* against some *Vibrio* pathogens and other bacteria as part of our exploration for new and novel bioactive compounds. 

## 2. Results and Discussion

The antibacterial activity of *Cocos nucifera* husk extract was investigated. Sixteen of the test bacteria were susceptible to the aqueous extract with a zone of inhibition value ranging between 11 and 20 mm, while twenty bacteria were susceptible to the *n*-hexane extract with inhibition zones ranging between 12 and 18 mm at the test concentration of 25 mg/mL (Table 1). Thirty-seven of the *Vibrio* isolates were susceptible, with inhibition zone diameters ranging between 10 and 18 mm for the aqueous extracts; while thirty-eight were susceptible to the *n*-hexane extract with inhibition zone diameters ranging between 12 and 21 mm (Table 2). The minimum inhibitory concentrations (MICs) of the extract against the susceptible bacteria generally ranged between 0.6–5.0 mg/mL for both extracts. Specifically, MICs for the aqueous and *n*-hexane extracts ranged between 0.6–5.0 mg/mL and 0.3–5.0 mg/mL respectively (Table 1). 

**Table 1:** Antibacterial activities of crude aqueous and *n*-hexane extracts of *C*. *nucifera* husk.

Isolate Identity	Inhibition zone (mm) / MIC			
	Aqueous extract	*n*-hexane extract	AMP	TET
*Escherichia coli* ATCC 8739	11 / 0.625	12 / 5.0	28	27
*Pseudomonas aeruginosa* ATCC 19582	- / ND	13 / 1.25	22	25
*Streptococcus faecalis ATCC* 29212	15 / 0.625	14 / 0.312	21	27
*Pseudomonas aeruginosa* ATCC 7700	16 / 2.5	18 / 2.5	15	20
*Klebsiella pneumoniae* ATCC 10031	22 / 1.25	15 / 2.5	21	30
*Klebsiella pneumoniae* ATCC 4352	13 / 1.25	- / ND	24	33
*Proteus vulgaris* CSIR 0030	20 / 1.25	16 / 1.25	24	35
*Bacillus subtilis* KZN	- / ND	12 / 2.5	28	22
*Pseudomonas aeruginosa* KZN	16 / 2.5	18 / 0.625	28	36
*Enterococcus faecalis* KZN	14 / 0.625	13 / 0.625	22	32
*Escherichia coli* KZN	13 / 1.25	12 / 2.5	26	38
*Staphylococcus aureus* KZN	- / ND	- / ND	27	36
*Staphylococcus aureus* OKOH1	14 / 2.5	14 / 0.625	23	34
*Staphylococcus aureus* OKOH2A	13 / 2.5	16 / 2.5	19	28
*Staphylococcus aureus* OKOH2B	- / ND	12 / 2.5	28	32
*Staphylococcus aureus* OKOH3	- / ND	- / ND	25	35
*Micrococcus kristinae*	- / ND	- / ND	22	32
*Serratia marscens* ATCC 9986	15 / 0.625	16 / 1.25	25	27
*A. calcaoceuticus anitratus* CSIR	- / ND	14 / 2.5	14	32
*Klebsiella pneumoniae* KZN	15 / 0.625	12 / 0.625	14	21
*Shigella flexineri* KZN	- / ND	15 /1.25	16	30
*Salmonella* specie KZN	15 / 1.25	14 / 2.5	17	25
*Staphylococcus epididirmis* KZN	18 / 0.625	12 / 0.625	17	18
*Micrococcus luteus*	14 / 1.25	12 / 0.625	24	33

**Key:** - represents no antibacterial activity; MIC represents minimum inhibitory concentration; ND represents not determined.

**Table 2:** Antivibriol activities of crude aqueous and n-hexane extracts of *C*. *nucifera* husk on *Vibrio* pathogens.

Isolate Identity	Inhibition zone (mm)/MIC			
	Aqueous Extract	*n*-hexane Extract	AMP	TET
*Vibrio vulnificus* EL047	17 / 0.625	18 / 0.625	25	18
*Vibrio* specie EL014	15 / 0.625	- / ND	26	16
*Vibrio* specie EL031	15 / 2.5	16 / 0.625	24	20
*Vibrio metschnkovii* EL003	- / ND	20 / 2.5	20	20
*Vibrio* specie EL006	16 / 1.25	15 / 2.5	16	20
*Vibrio fluvialis* EL049	14/0.625	- / ND	20	19
*Vibrio* specie EL027	13 / 1.25	- / ND	40	22
*Vibrio* specie EL052	16 / 1.25	17 / 1.25	15	32
*Vibrio fluvialis* EL007	16 / 2.5	18 / 0.625	15	29
*Vibrio vulnificus* EL051	13 / 0.625	- / ND	27	17
*Vibrio fluvialis* EL036	14 / 1.25	12 / 2.5	14	30
*Vibrio fluvialis* EL015	- / ND	15 / 1.25	16	18
*Vibrio vulnificus* EL017	15 / 2.5	16 / 0.625	17	18
*Vibrio* specie EL013	16 / 2.5	18 / 2.5	28	17
*Vibrio metschnkovii* EL028	18 / 1.25	14 / 1.25	22	16
*Vibrio vulnificus* EL039	/0.625	- / ND	30	21
*Vibrio metschnkovii* EL008	14 / 0.625	12 / 1.25	19	28
*Vibrio fluvialis* EL035**	13 / 1.25	12 / 0.625	12	26
*Vibrio vulnificus* EL002	- / ND	12 / 1.25	15	30
*Vibrio vulnificus* EL005	16 / 0.625	18 / 0.625	13	15
*Vibrio* specie EL021	16 / 2.5	20 / 2.5	20	29
*Vibrio vulnificus* EL018	- / ND	21 / 2.5	21	20
*Vibrio vulnificus* EL043	10 / 0.625	- / ND	12	18
*Vibrio vulnificus* EL045	18 / 1.25	12 / 1.25	15	28
*Vibrio parahaemolyticus* AL045	14 / 1.25	15 / 1.25	16	18
*Vibrio vulnificus* EL040	12 / 2.5	16 / 0.625	20	16
*Vibrio vulnificus* EL012	14 / 1.25	12 / 2.5	19	17
*Vibrio fluvialis* EL034	- / ND	14 / 0.625	12	18
*Vibrio vulnificus* EL044	15 / 2.5	18 / 2.5	16	16
*Vibrio vulnificus* EL053	- / ND	16 / 2.5	12	40
*Vibrio fluvialis* EL042	18 / 1.25	18 / 1.25	26	40
*Vibrio fluvialis* EL041	15 / 0.625	18 / 0.625	28	40
*Vibrio vulnificus* EL048	15 / 0.625	21 / 0.625	21	34
*Vibrio vulnificus* EL050	16 / 2.5	18 / 0.625	22	35
*Vibrio vulnificus* EL010	13 / 0.625	15 / 1.25	21	35
*Vibrio* specie EL009	13 / 5.0	17 / 2.5	20	21
*Vibrio* specie AL046	13 / 2.5	13 / 2.5	22	20
*Vibrio* specie EL054**	15 / 1.25	13 / 1.25	22	40
*Vibrio vulnificus* EL036	13 / 1.25	16 / 1.25	24	38
*Vibrio vulnificus* EL039	14 / 2.5	18 / 0.625	24	26
*Vibrio vulnificus* EL033	- / ND	14 / 0.625	13	18
*V. fluvialis* AL019	14 / 1.25	12 / 2.5	12	29
*V. fluvialis* EL032	18 / 1.25	18 / 0.625	30	32

Key: - represents no antibacterial activity; MIC represents minimum inhibitory concentration; ND represents not determined, AMP – ampicillin, TET – tetracycline.

The results of time-kill studies are presented in Table 3. Data are presented in terms of the Log_10_ cfu/mL reduction in viable cell count and interpretations are based on the conventional bactericidal activity standard, which is, a 3 Log_10_ cfu/mL or greater reduction in the viable colony count [15]. For the aqueous extract, average log reduction in viable cell count in time kill assay ranged between 0.12 Log_10_ and 4.2 Log_10_ cfu/mL after 8 hours interaction at 1 × MIC and 2 × MIC. For the *n*-hexane extract, the log reduction ranged between 0.56 Log_10_ and 6.4 Log_10_ cfu/mL after 8 hours interaction in 1 × MIC and 2 × MIC. The greatest reductions in cell density achieved with the aqueous extract were on *Vibrio vulnificus* EL039, with the average value of 4.2 Log_10_ cfu/mL, *S. aureus* OKOH2B (clinical strain) with the average reduction in viable cell count of 3.46 log_10_ cfu/mL, while the greatest reduction in viable cell volume achieved by the *n*-hexane extract were on the environmental strain *Bacillus substilis*, with the average value of 6.40 log_10_ cfu/mL and the reference strain *Escherichia coli* ATCC 8739, with an average reduction in viable cell count of 5.6 log_10_ cfu/mL.

The crude aqueous extract was bactericidal against *B. substilis, V. vulnificus* EL039 *and V. fluvialis* EL041 at 1 × MIC and 2 × MIC after an 8 h interaction period and bacteriostatic during the first 4 h of interaction at both MIC levels, while the *n*-hexane extract was bactericidal against nine of the test bacteria: *E. coli, A. calcaoceticus anitratus* CSIR, *Staphylococcus aureus* clinical strain*, B. substilis* environmental strain and the *Vibrio strains V. metschnkovii* EL008, *V. specie* EL009, *V. vulnificus* EL039 *and V. fluvialis* at both MIC levels after 8 h of interaction, but bacteriostatic against *S. faecalis* ATCC 29212 after 8 h of interaction. 

**Table 3:** Nature of inhibition of crude aqueous and *n*-hexane extracts of *C. nucifera* husk against some bacterial isolates and *Vibrio* pathogens.

Susceptible isolate	Aqueous extract				
	MIC (mg/mL)	Log_10_ Kill (MIC)		Log_10_ Kill (2*MIC)	
		4 h	8 h	4 h	8 h
*Vibrio metschnkovii* EL008	0.625	2.0	2.2	2.6	2.4
*Vibrio specie* EL009	5.0	1.2	2.4	2.0	2.2
*Vibrio vulnificus* EL039	0.625	2.4	2.8	2.6	4.2*
*Vibrio fluvialis* EL041	0.625	1.8	2.4	2.4	3.4*
*Escherichia coli* ATCC8739	0.625	0.64	0.72	0.70	0.92
*Streptococcus faecalis* ATCC 29212	0.625	0.12	0.48	1.24	1.48
*Acinetobacter calcaoceticus* anitratus CSIR.	NA	NA	NA	NA	NA
*Bacillus substilis* ^∞^	NA	NA	NA	NA	NA
*Shigella flexineri* ^∞^	NA	NA	NA	NA	NA
*Staphylococcus aureus*	0.625	1.40	1.62	2.21	3.46*
	*n*-hexane extract				
*Vibrio metschnkovii* EL008	1.25	3.0*	3.4*	2.8	3.2*
*Vibrio specie* EL009	2.5	3.4*	3.8*	4.0*	4.2*
*Vibrio vulnificus* EL039	0.625	3.2*	4.0*	4.2*	4.2*
*Vibrio fluvialis* EL041	0.625	4.0*	4.6*	4.2*	5.0*
*Escherichia coli* ATCC8739	5.0	3.5*	5.6*	5.6*	5.6*
*Streptococcus faecalis* ATCC 29212	0.312	1.02	1.28	1.22	2.46
*Acinetobacter calcaoceticus* anitratus CSIR.	2.5	2.1	2.2	4.2*	4.2*
*Bacillus substilis* ^∞^	2.5	0.56	4.22*	2.84	6.40*
*Shigella flexineri* ^∞^	1.25	4.2*	4.3*	4.3*	4.3*
*Staphylococcus aureus*	1.25	1.20	2.40	2.04	3.40*

**Key:** MIC represents minimum inhibitory concentration; * represents bactericidal effect; NA represents no activity; α represent clinical strains; ∞ represent environmental strains.

The use of plant extracts with medicinal potential represents a valid alternative for the treatment of different ailments and diseases. The antivibrio and antibacterial properties of the husk of *C. nucifera* were investigated against a number of *Vibrio* pathogens and other bacteria pursuant to contributing to our body of knowledge on the potentials o the plant in the management of *Vibrio* and other bacterial infections in support of previous report [16]. The aqueous and *n*-hexane extracts of the husk of our study plant exhibited potent antivibrio and antibacterial activity against about 90% of the bacteria strains tested. The result from this study confirms that both the aqueous and *n*-hexane fraction of the husk possess antimicrobial properties against Vibrio species and other bacteria thus supporting the traditional use of this plant in the treatment of wound, respiratory and gastro intestinal tract infections. 

The diameters of the zones of inhibition exhibited by the extracts against the text bacteria are similar to those reported elsewhere, *viz*. by Ravikumar *et al.* [17] and Chandrasekaran *et al.* [18], who reported on the chloroform extracts of *Exoecaria agallocha* leaves; as well as methanol and aqueous extracts of mangrove respectively. The limited activity of the aqueous extract in comparison to the n-Hexane extract corroborate previous reports [19,20] where they reported lower activity in the aqueous extracts compared to other solvent extracts. 

The MICs values observed in this study varied depending on the strain and ranged from 0.6 to 5.0 mg/mL for the *Vibrio* bacteria and from 0.3 to 5.0 mg/mL for the other bacterial isolates. The observation that some of the *Vibrio* and bacteria strains were susceptible to the plant extract at a concentration as low as 0.3125 mg/mL strongly suggest that of *C. nucifera* plant can be effective in the treatment of infections caused by these pathogens. A similar result was reported by Sharma *et al.* [21] in their studies on the vibriocidal activities of 16 Indian medicinal plants, wherein 70% of the *Vibrio* pathogens tested were susceptible to the plant extract at a concentration ranging between 2.5 and 20 mg/mL. 

The bactericidal activities of the aqueous extracts of this plant at 2 × MIC after 8 h exposure against *S. aureus* OKOH2B (a clinical isolate from wound sepsis); *V. vulnificus* and *V. fluvialis* is worth noting and further supports its use as a folklore remedy. At 1 × MIC, the *n*-hexane extract showed bactericidal activity against three of the six bacterial species tested as well as the entire cohort of *Vibrio* isolates. At 2 × MIC the entire population of the *Vibrio* and the other bacteria species (except *S. faecalis*) tested had been wiped out after 8 hr exposure. It would appear that the observed bactericidal or bacteriostatic activity of this plant is both time and concentration dependent. 

Considering the crude nature and low toxicities of the solvent extracts used in this study, our results allow us to conclude that the crude extract from *C. nucifera* exhibited significant antibacterial activity and properties that support its folkloric use in the treatment of some food borne diseases as well as its potential wound healing activities. Plants that have tannins as their components are astringent in nature and are used for treating intestinal disorder such as dysentery and diarrhea [22] thus exhibiting antimicrobial activity. Esquenazi *et al*. [11] reported that *C. nucifera* aqueous extract is rich in catechin and epicatechin together with condensed tannin. Edeoga *et al*. [23] had reported that the curative potentials of plants are locked-up and embedded in some chemical components that effect physiological responses in man. Some of these ingredients act synergistically to confer bioactivity on a plant an active material.

To further buttress the phytochemical importance of *C. nucifera,* Zakaria *et al.* [24] administered the coconut juice extract as part of a dietary supplement at low concentrations and also the coconut cream and oil. The application of *C. nucifera* extract as food supplement is both an economical and an eco-friendly alternative in antimicrobial chemotherapy. Although coconut fruit is meant for human consumptions, this present study suggests the need for characterizing the antibacterial active principle(s) of *Cocos nucifera*. Understanding the chemical nature of the active principle(s), it will provide an opportunity to synthesize new and effective antibacterial (including antivibrio) drugs. 

## 3. Experimental

### 3.1. Plant material

The plant specimens were collected from the vicinity of the Research Farm of the Obafemi Awolowo University, Ile Ife, Nigeria and identified by the curator of the Herbarium at the Department of Botany, Obafemi Awolowo University, and a voucher specimen kept there.

### 3.2. Preparation of extracts

The coconut husk was sun–dried, milled and sieved manually to obtain fine powdered particles. About 50 g dried of powdered husk of the plant was extracted at room temperature and for 48 h with 95% n-hexane (200 mL) using a Soxhlet extraction method. The mixture was then filtered using Whatman No. 1 filter paper. The filtrates of each extraction were pooled together and concentrated to dryness *in vacuo* using a rotary evaporator to remove the *n*-hexane. The concentrated extract was then allowed to dry at room temperature to a constant weight. For the aqueous extract, about 50 g of the powdered extract was dissolved in sterile distilled water (500 mL) for 24 h with shaking. The resulting extracts were centrifuged at 3,000 rpm for 5 min at 4 °C. The supernatant was filtered through a Whatman No. 1 filter paper and the filtrate was lyophilized. 

### 3.3. Test bacterial strains

The bacterial isolates used in this study included forty-five *Vibrio* strains and twenty-five bacteria pathogens as part of the culture collection of the Applied and Environmental Microbiology Research Group (AEMREG), Department of Biochemistry and Microbiology, University of Fort Hare, Alice, South Africa. The *Vibrio* species were isolated from waste-water effluent in the Eastern Cape Province, South Africa. The bacterial isolates include reference strains (9) obtained from the South African Bureau of Standard (SABS), environmental strains (12) and clinical isolates (4).

### 3.4. Antibacterial susceptibility test

The susceptibility screening of the test bacteria to both crude extracts and standard antibiotics were done in accordance with the methods described elsewhere [25,26]. The inoculum size of each test strain was standardized at 5 × 10^5^ cfu/mL using McFarland Nephelometer standard. Sterile Mueller-Hinton agar plates were seeded with test bacterial strains and allowed to stand at 37 °C for 3 h. Wells were then bored into the agar media using a sterile 6 mm cork borer and the wells filled with the solution of the extracts and antibiotics taking care not to allow spillage of the solution onto the surface of the agar. The plates were allowed to stand on the laboratory bench for 1 h to allow proper diffusion of the extract and antibiotics into the media and thereafter incubated at 37 °C for 24 h, after which they were observed for zones of inhibition. The effects of the extracts on the test bacterial isolates were compared with those of the standard antibiotics tetracycline and ampicillin which served as a negative and positive control at a concentration of 1 mg/mL and 10 μg/mL, respectively.

### 3.5. Determination of minimum inhibitory concentration (MIC)

The MIC of the crude aqueous and *n*-hexane extract was carried out using the method of Akinpelu and Kolawole [27]. Two-fold dilutions of the extracts were prepared and 2 mL aliquot of different concentrations of the solution were added to 18 mL of pre-sterilized molten Mueller-Hinton agar at 40 °C to give final concentration regimes of 5.0 to 0.156 mg/mL. The medium was then poured into sterile Petri dishes and allowed to set. The surfaces of the media were allowed to dry under a laminar flow before streaking with 18 h old bacterial cultures. The plates were later incubated at 37 °C for up to 72 h after which they were examined for the presence or absence of growth. The MIC was taken as the least concentration of extracts that will prevent the visible growth of the test bacteria.

### 3.6. Time-kill assay

Determination of the kill rate of the crude extracts was done following the procedure as described by Okoli and Iroegbu [28]. Inocula were prepared following the described guidelines of EUCAST [29]. The resultant suspension were diluted 1:100 with fresh sterile broth and used to inoculate 50 mL volumes of Mueller Hinton broth incorporated with extracts at MIC and 2 × MIC to a final cell density of approximately 5 × 10^5^ cfu/mL. The flasks were incubated at 37 °C on an orbital shaker at 120 rpm. A 500 μL sample was removed from cultures at 0, 4 and 8 h, diluted serially and 100 μL of the diluted samples were plated on Mueller Hinton agar plates and incubated at 37 °C for 24 h. Controls included extract-free Mueller Hinton broth seeded with the test inoculum.

## 4. Conclusion

This study has demonstrated the antibacterial activities of *C. nucifera* especially against *Vibrio* bacteria and suggests that the plant has immense potentials as an alternative to synthetic antibiotics in the management of *Vibrio* and other bacterial infections. Further studies are needed to elucidate the active components and their modes of action as well as their potentials in combination chemotherapywith synthetic drugs which is the subject of ongoing research in our group.
